# The prognostic significance of MRI-detected extramural venous invasion, mesorectal extension, and lymph node status in clinical T3 mid-low rectal cancer

**DOI:** 10.1038/s41598-019-47466-0

**Published:** 2019-08-29

**Authors:** Chaoyang Gu, Xuyang Yang, Xubing Zhang, Erliang Zheng, Xiangbing Deng, Tao Hu, Qingbin Wu, Liang Bi, Bing Wu, Minggang Su, Ziqiang Wang

**Affiliations:** 1 Department of Gastrointestinal Surgery, West China Hospital, Sichuan University, No. 37 Guo Xue Alley, Chengdu, 610041 Sichuan Province China; 2 Department of Radiology, West China Hospital, Sichuan University, No. 37 Guo Xue Alley, Chengdu, 610041 Sichuan Province China

**Keywords:** Gastrointestinal cancer, Outcomes research, Cancer therapy

## Abstract

The purpose of this study was to evaluate the prognostic significance of the magnetic resonance imaging-detected extramural venous invasion (MR-EMVI), the depth of mesorectal extension (MR-DME), and lymph node status (MR-LN) in clinical T3 mid-low rectal cancer. One hundred and forty-six patients with clinical T3 mid-low rectal cancer underwent curative surgery were identified. Pretreatment high-resolution MRI was independently reviewed by two experienced radiologists to evaluate MR-EMVI score (0–4), MR-DME (≤4 mm or >4 mm), and MR-LN (positive or negative). The Cox-multivariate regression analysis revealed that the MR-EMVI was the only independent prognostic factor that correlated with overall 3-year disease-free survival (DFS) (p = 0.01). The survival analysis showed that patients with positive MR-EMVI, MR-DME > 4 mm, and positive MR-LN had a poorer prognosis in the overall 3-year DFS (HR 3.557, 95% CI 2.028 to 13.32, p < 0.01; HR 3.744, 95% CI:1.165 to 5.992, p = 0.002; HR 2.946, 95% CI: 1.386 to 6.699, p < 0.01). By combining MR-EMVI with MR-DME or MR-LN, the prognostic significance was more remarkable. Our study suggested that the MR-EMVI, MR-DME, and MR-LN were the important prognostic factors for patients with clinical T3 mid-low rectal cancer and the MR-EMVI was an independent prognostic factor.

## Introduction

Rectal cancer is one of the most prevalent cancers worldwide and an important cause of cancer mortality for both sexes^1^. The depth of infiltration of the primary tumor, lymph node status, and extramural venous invasion (EMVI) on the traditionally histopathological examination were well-known as important prognostic factors for local recurrence and distant metastasis in patients with rectal cancer. Previous studies suggested that venous invasion detected in the histopathological examination was associated with a higher incidence of local or distant recurrence^2–7^. Similarly, the depth of mesorectal extension (DME) was also correlated with prognosis and should be considered in therapeutic decision making^8–16^.

However, pathological EMVI and DME can only be confirmed after operation. With the development of magnetic resonance imaging (MRI) technology, high-resolution MRI had become the standard measurement tool in accurately evaluating DME for its consistency with pathological results^17,18^. According to the identification of expanded vessels or tumor signals in the venous lumen^17^, MRI can preoperatively detect EMVI in rectal cancer patients^17,19^. Additionally, for the local staging of rectal cancer, MRI is more reliable than computed tomography (CT) and endoluminal ultrasound. The local staging of rectal carcinoma with high-resolution MRI had been shown to be accurate^17,20^. It plays an important role in the preoperative planning of primary tumor resection and indicating the need for neoadjuvant therapy. The aim of this detailed preoperative staging was to facilitate long-term disease-free survival.

To the best of our knowledge, however, there are few studies combining MR-EMVI, MR-DME, and MR-LN to predict the prognosis of clinical T3 mid-low rectal cancer. The purpose of present work is to explore the prognostic significance of MRI-detected extramural venous invasion, mesorectal extension, and lymph node status in clinical T3 mid-low rectal cancer.

## Materials and Methods

### Patients

**Figure 1:**
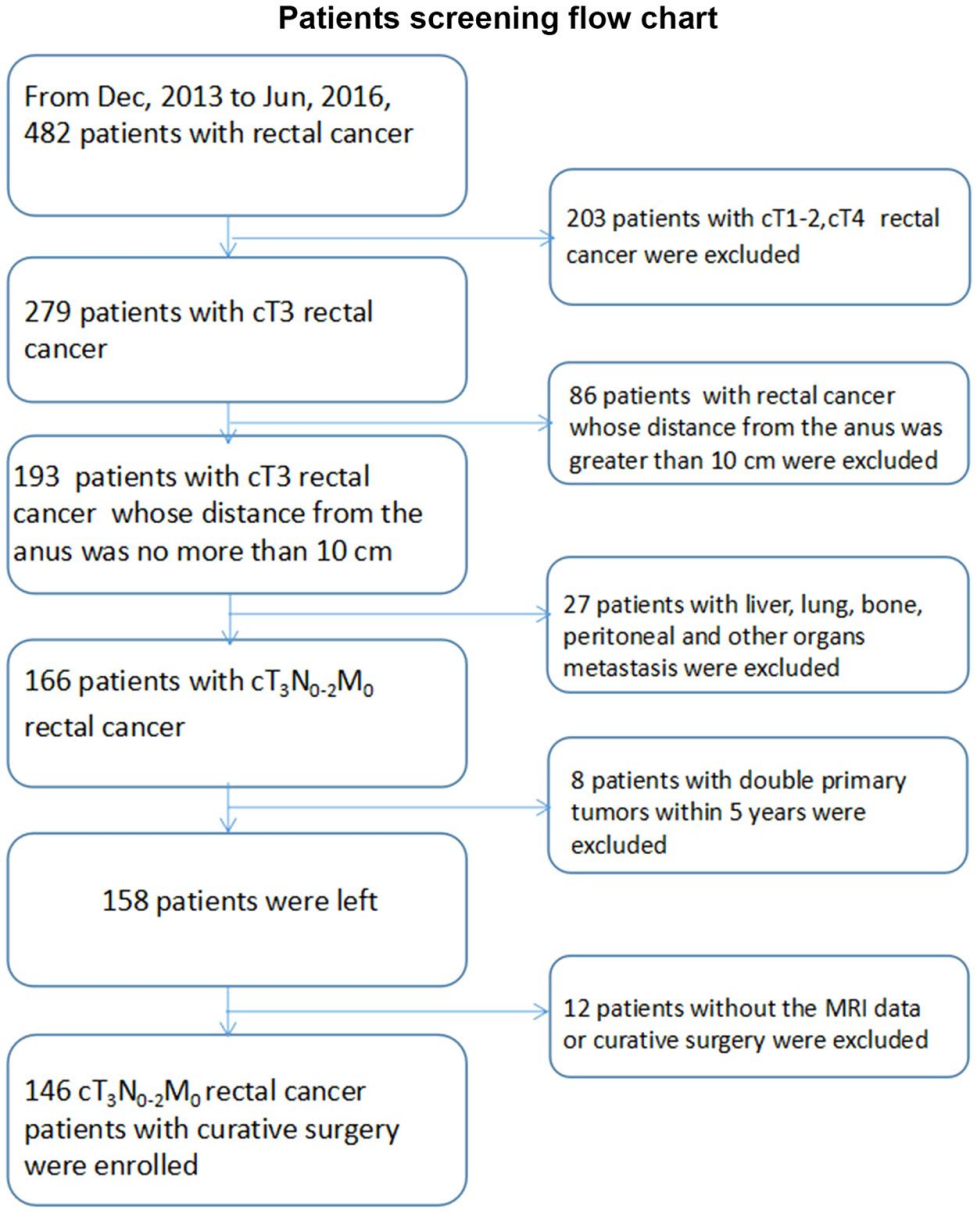
Patients screening flow chart.

Patients with mid-low rectal cancer underwent curative resection between December 2013 and June 2016 in our hospital were identified. Based on the distance from tumor lower margin to anal verge measured by rigid sigmoidoscopy, the primary rectal tumor was categorized as low (up to 5 cm), middle (from >5 to 10 cm) or high (from >10 up to 15 cm). Patients were excluded if any of the following criteria were met: clinical T1/T2/T4 tumor before any treatment; high rectal cancer; primary tumor with synchronous distant metastasis; the second malignancies occurred within 5 years after the primary operation; no available or incomplete MRI information before any treatment. The patients screening flow chart was shown in Fig. 1. Patients’ demographic, clinicopathological characteristics, and MRI data (before neoadjuvant chemoradiotherapy, nCRT) were collected from the prospective database. Approval from the Ethics Committee of our hospital was obtained and the Ethics Committee had agreed with the request for waiver of informed consent.

### Imaging before any treatment

After admission, patients accepted examination including enteroscopy, blood test, and CT scan. A 3.0 T MRI (GE Discovery MR750W) using a phased-array body coil was imaged for each patient. The standard imaging protocol includes a sagittal T2 weighted (T2W) fast spin echo and an oblique axial thin-section T2W (TR: 4000 TE: 100; SLICE: 3 mm; MATRIX: 256 × 256; FOV: 16; Plane resolution: 0.5–0.8 mm). Patients need to empty the rectum with Suppositories Glycerol and inject antispasmodic medication to inhibit bowel peristalsis in 30 minutes before the MR examinations. The MRI data (EMVI, DME, LN) were analyzed independently by two radiologists (with more than 10 years of experience in MRI) who were blind to clinicopathological findings. Any discrepancy was solved by discussion.

### Interpretation of features detected on the high-resolution MRI

#### MR-EMVI criteria

**Figure 2:**
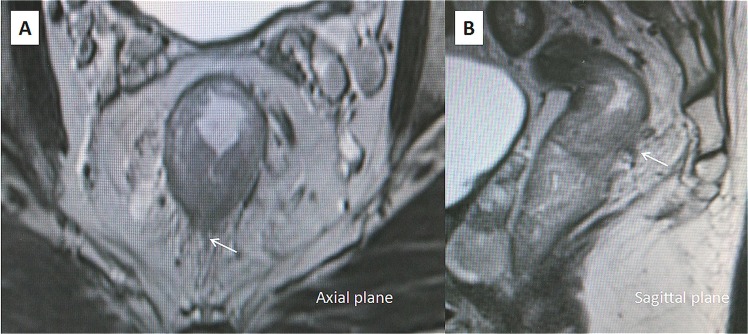
T2-weighted axial (**A**) and sagittal (**B**) magnetic resonance images. Magnetic resonance imaging-detected EMVI (MR-EMVI) score = 3: intermediate tumor signal intensity apparent within vessels with caliber slightly expanded (the white arrow).

**Figure 3:**
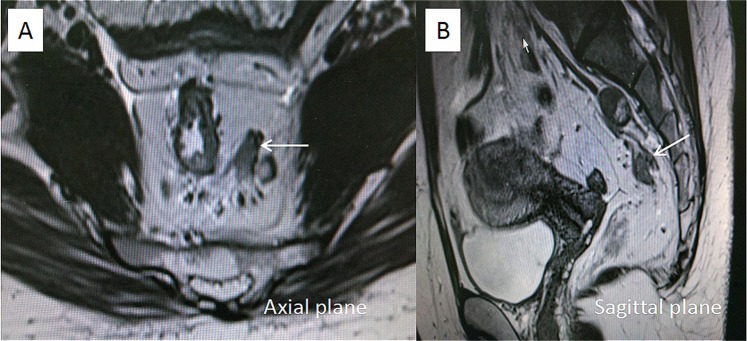
T2-weighted axial (**A**) and sagittal (**B**) magnetic resonance images. Magnetic resonance imaging-detected EMVI (MR-EMVI) score = 4: an obvious irregular vessel with definite tumor signal (the white arrow).

According to the system proposed by Smith, the EMVI grading score was adopted^21^. Grade 0–2 was identified as negative disease and were recorded without distinction. Grades 3 and 4 were defined as EMVI-positive disease. Grade 3 (Fig. 2) EMVI describes intermediate signal intensity apparent within vessels, although the contour and caliber of these vessels are only slightly expanded. Grade 4 (Fig. 3) EMVI describes obvious irregular vessel contour or nodular expansion of vessel by definite tumor signal.

#### MR-DME criteria

**Figure 4:**
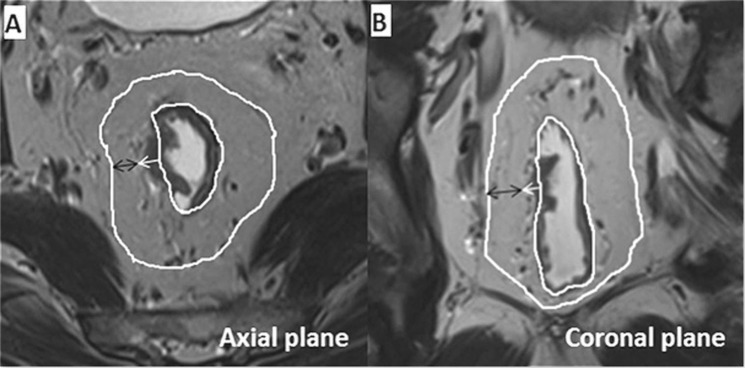
T2-weighted axial (**A**) and coronal (**B**) magnetic resonance images. Magnetic resonance imaging-detected the depth of mesorectal extension (MR-DME) ≤ 4 mm and negative MRF. The black double-headed arrow indicates the minimum distance from the tumor to the mesorectal fascia, and the white single-headed arrow indicates the MR-DME. The white line indicates the mesorectal fascia and the muscularis propria.

**Figure 5:**
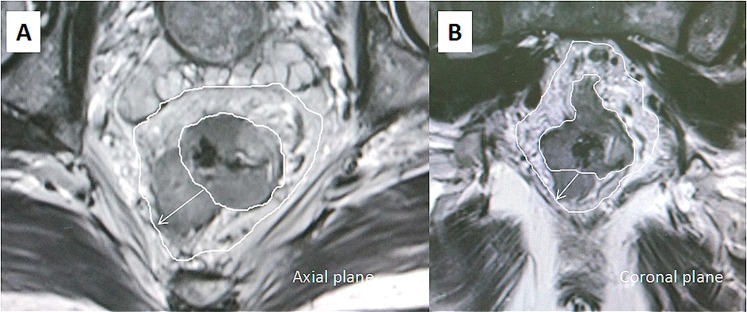
T2-weighted axial (**A**) and coronal (**B**) magnetic resonance images. Magnetic resonance imaging-detected the depth of mesorectal extension (MR-DME) > 4 mm and positive MRF.

The method of measurement of DME was adopted from Toshinori^22^. The DME was measured as the distance from the outer margin of the muscular layer to the deepest site of the tumor extension (in millimeters). When the outer margin of the muscular layer can’t be identified entirely, the outer boundary was estimated by depicting a straight line between the two breakpoints of the muscular layer (Figs 4 and 5).

#### MR-LN criteria

**Figure 6:**
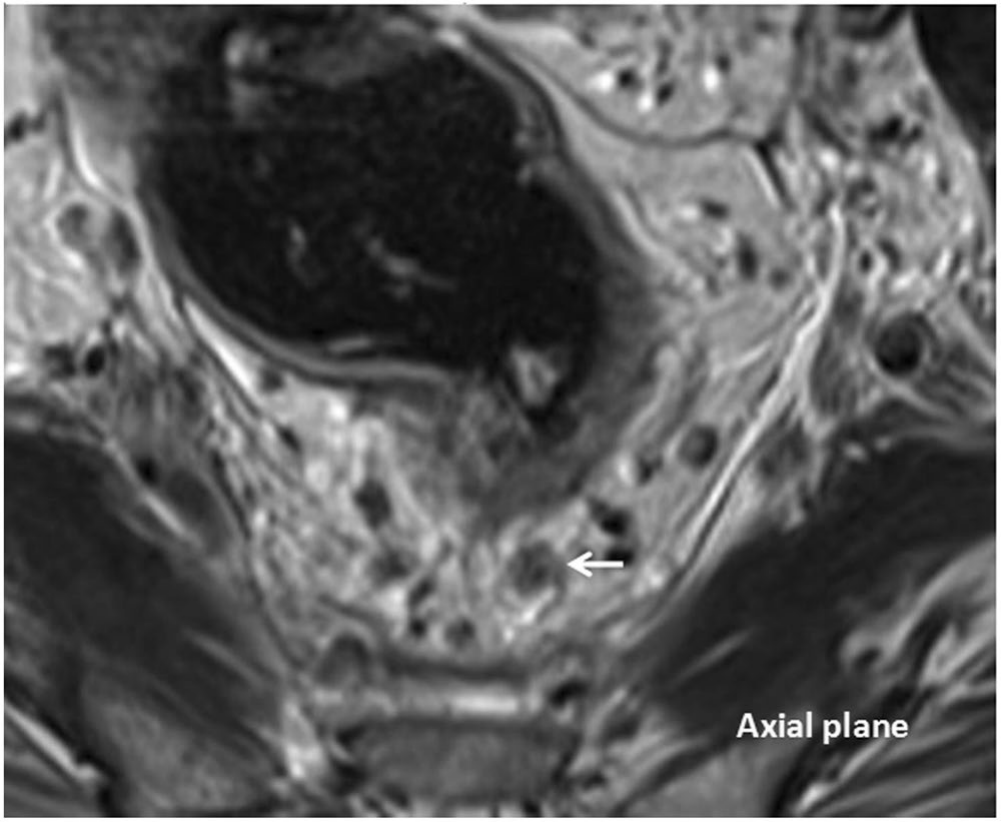
T2-weighted axial magnetic resonance images. Magnetic resonance imaging–detected positive lymph node (MR-LN): the short diameter of this lymph node is greater than 5 millimeters and the border is irregular and the signal is heterogeneous (the white arrow).

Lymph nodes on MRI were considered as positive if any of the following characteristics were present: (1) the largest short-axis diameter greater than 5 mm^23,24^; (2) irregular border or heterogeneous signal^25^ (Fig. 6).

### The treatment strategy and follow-up

After the initial staging, the multidisciplinary treatment team (MDT) would decide whether neoadjuvant therapy was needed or not, which mainly depended on EMVI score, lymph node status, mesorectal fascia(MRF) involved or not, and the depth of tumor invasion. nCRT regimen usually includes short-course radiotherapy (5 × 5 Gy) and 5-FU based long-course chemoradiotherapy (45–50.4 Gy). Patients with short-course radiation therapy will be performed curative resection within 7 days compared with long-course nCRT and surgery within 6 to 8 weeks. Fluorouracil-based postoperative chemotherapy with or without radiotherapy was performed for all pathological stage III patients and stage II patients with pathological high-risk factors. The follow-up schedule after the resection was based on the guideline of the National Comprehensive Cancer Network (NCCN) of the Rectal Cancer^26^, which was described in our previous study^27^.

### Statistical analysis

All statistical analyses were performed using the IBM SPSS version 20.0 (IBM Inc., Armonk, NY). p < 0.05 was considered as statistical significance. The primary outcomes were 3-year disease-free survival (DFS) and 3-year overall survival (OS). The DFS was defined as the duration from surgery to any relapse or all-cause death. The OS refers to the duration from the first diagnosis to any cause that causes death. Ten clinical independent factors before operation were analyzed. Survival analysis was performed by using the Kaplan-Meier method, groups were compared using the log-rank test. The Cox regression analysis was also used to identify the preoperative independent prognostic factors for the DFS and OS, risk factors for p < 0.05 in the univariate analysis will be included in the multivariate analysis.

## Results

Table 1 showed the patients’ baseline characteristics. The male to female was 95:51. The median age was 61.5 years (range 32–87). Of the 146 patients, MR-EMVI with score 0–2 was found in 109 patients and MR-EMVI with score 3–4 was found in 37 patients. MR-DME > 4 mm was found in 99 patients and MR-DME ≤ 4 mm was found in 47 patients. Positive MR-LN was found in 60 patients and negative MR-LN was found in 86 patients.

**Table 1:** Patient baseline characteristics (n = 146).

characteristics	No. of patients (%)
Gender	
Female	51 (34.9)
Male	95 (65.1)
Age (y)	
<65	86 (41.1)
>=65	60 (58.9)
median(range)	61 (32–87)
Distance from anal verge (cm)	
<=5	58 (39.7)
<=10	88 (60.3)
Neoadjuvant therapy	
Yes	58 (39.7)
No	88 (60.3)
Adjuvant therapy	
Yes	70 (47.9)
No	76 (52.1)
Preoperative serrum CEA level (ng/ml)	
<5	92 (63.0)
>=5	54 (37.0)
Preoperative serum CA19-9 level (U/ml)	
<37	131 (89.7)
>=37	15 (10.3)
Resection type	
Elape + Miles + Hartmann	29 (19.9)
ISR + Dixon	117 (80.1)
Combined resection	
Yes	10 (6.8)
No	136 (93.2)
Differentiation	
G1 + G2	83 (56.9)
G3 + G4	63 (43.2)
Pathological lymphatic invasion	
Yes	55 (37.7)
No	91 (62.3)
Pathological tumor size (cm)	
<5	121 (82.9)
>=5	25 (7.1)
Pathological venous invasion	
Yes	15 (10.3)
No	131 (89.7)
Pathological T stage	
T2	6 (4.1)
T3 + T4	132 (90.4) + 8 (5.5)
CRM	
Positive	0(0.0)
Negative	146(100.0)
MR-DME	
<=4 mm	47 (32.2)
>4 mm	99 (67.8)
MRF	
Positive	29 (19.9)
Negative	117 (80.1)
MR-LN	
Positive	60 (41.1)
Negative	86 (58.9)
MR-EMVI score	
0–2	109 (74.7)
3–4	37 (25.3)

Elape: Extralevator abdominoperineal excision.

ISR: Intersphincteric resection.

CRM: Circumferential Resection Margin.

MR-DME: Magnetic Resonance Imaging -detected depth of mesorectal extension.

MRF: Mesorectal Fasica.

MR-LN: Magnetic Resonance Imaging -detected lymph node.

MR-EMVI: Magnetic Resonance Imaging -detected extramural venous invasion.

Furthermore, MR-EMVI score ≥ 3 and MR-DME > 4 mm were found in 42 patients a. MR-EMVI score ≤ 2 and MR-DME ≤ 4 mm were found in 32 patients. In addition, MR-EMVI score ≥ 3 and Positive MR-LN was found in 24 patients. MR-EMVI score ≤ 2 and negative MR-LN were found in 74 patients. Combined resection with suspected invaded organs was performed in 10 of 146 patients (6.8%) (three with the ovary, one with the internal iliac vessels, four with the seminal vesicles, one with the neurovascular bundle, and two with the posterior wall of bladder). The R0 resection rate was 100 percent. No circumferential resection margin (CRM) involvement was found in all pathological specimens. Neoadjuvant chemotherapy with or without radiotherapy was performed in 58 patients.

The median follow-up was 32 months (range 21–51). The overall local recurrence occurred in 5 of 146 patients (3.4%) and the distant metastasis occurred in 23 of 146 patients (15.8%). The median time of distant metastasis was 12 months (range 3–43). The 3-year DFS rate was 84.9% in overall population, 60.3% in patients with MR-EMVI score ≥ 3, 86.2% in patients with MR-EMVI score ≤ 2, 75.7% in patients with MR-DME > 4 mm, 89.8% in the MR-DME ≤ 4 mm patients, 69.9% in patients with positive MR-LN, and 87.2% in patients with negative MR-LN. The 3-year OS rate was 95.6% in overall population, 87.8% in patients with MR-EMVI score ≥ 3, 96.3% in patients with MR-EMVI score ≤ 2, 92.7% in patients with the MR-DME > 4 mm, 100.0% in patients with MR-DME ≤ 4 mm, 89.1% in patients with positive MR-LN, and 98.8% in patients with negative MR-LN.

**Figure 7:**
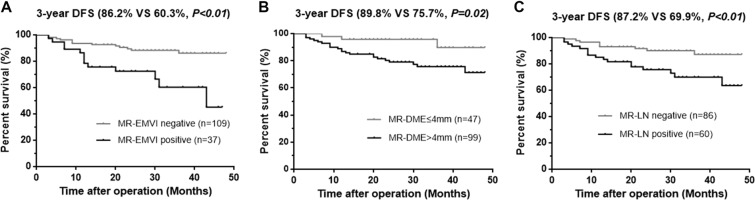
Kaplan - Meier analysis for the DFS according to MRI-detected factors. (**A**) The 3-year DFS rate was 60.3% in patients with positive MR-EMVI (MR-EMVI score ≥ 3)and 86.2% in patients with negative MR-EMVI (MR-EMVI score ≤ 2). (**B**) The 3-year DFS rate was 75.7% in patients with the MR-DME > 4 mm and 89.8% in the patients with MR-DME ≤ 4 mm. (**C**) The 3-year DFS rate was 69.9% in patients with positive MR-LN, and 87.2% in patients with negative MR-LN.

**Figure 8:**
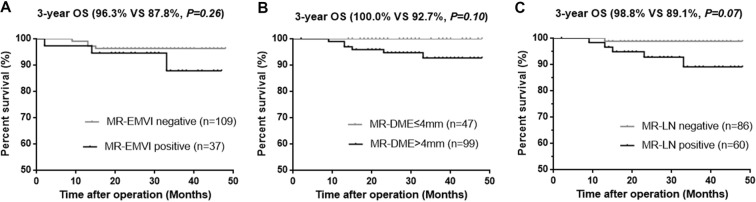
Kaplan - Meier analysis for the OS according to MRI-detected factors. (**A**) The 3-year OS rate was 87.8% in patients with positive MR-EMVI (MR-EMVI score ≥ 3) and 96.3% in patients with negative MR-EMVI (MR-EMVI score ≤ 2). (**B**) The 3-year OS rate was 92.7% in patients with MR-DME > 4 mm and 100.0% in patient with MR-DME ≤ 4 mm. (**C**) The 3-year OS rate was 89.1% in patients with positive MR-LN, and 98.8% in patients with negative MR-LN.

**Figure 9:**
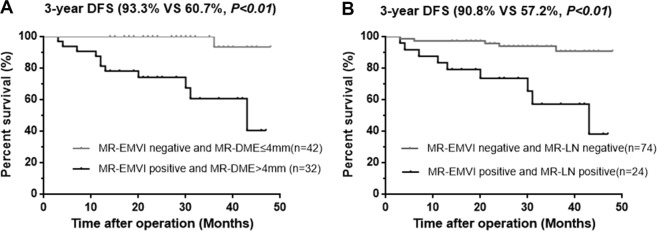
Kaplan - Meier analysis for the DFS according to MRI-detected factors. (**A**) The 3-year DFS rate was 60.7% in patients with positive MR-EMVI (MR-EMVI score ≥ 3) and MR-DME > 4 mm, and 93.3% in patients with negative MR-EMVI (MR-EMVI score ≤ 2)and MR-DME ≤ 4 mm. (**B**) The 3-year DFS rate was 57.2% in patients with positive MR-EMVI (MR-EMVI score ≥ 3) and positive MR-LN, and 90.8% in patients with negative MR-EMVI (MR-EMVI score ≤ 2) and negative MR-LN.

The results of the univariate and multivariate analysis were shown in Table 2, and the results of survival analysis according to MRI-detected factors were shown in Table 3. Patients with MR-EMVI score ≥ 3 had a significantly poorer prognosis in the 3-year DFS (60.3% VS 86.2%, p < 0.01) (Fig. 7A). A poorer prognosis of patients with a MR-DME > 4 mm was also observed in the 3-year DFS (75.7% VS 89.8%, *p* = 0.031) (Fig. 7B). Patients with positive MR-LN also had a significantly poorer prognosis in the 3-year DFS (69.9% VS 87.2%, *p* < 0.01) (Fig. 7C). Although the 3-year overall survival of patients with three prognostic factors did not reach statistical significance, there was a trend to poor prognosis (Fig. 8A–C). Furthermore, patients with MR-EMVI score ≥ 3 and MR-DME > 4 mm had a significantly poorer prognosis than patients with MR-EMVI score ≤ 2 and MR-DME ≤ 4 mm in the DFS (60.7% VS 93.3%, *p* < 0.01) (Fig. 9A). Similarly, patients with MR-EMVI score ≥ 3 and positive MR-LN had a poorer prognosis than those with MR-EMVI score ≤ 3 and negative MR-LN (57.2% VS 90.8%, p < 0.01) (Fig. 9B). In the multivariate analysis, MR-EMVI was the only independent significant factor that correlated with overall 3-year DFS (HR:3.236 95%CI: 1.328–7.885, *p* = 0.01).

**Table 2:** Correlation between preoperative clinical factors and DFS, and OS in clinical T3 mid-low rectal cancer.

Characteristics	N	DFS				OS			
		Univariate	Multivariate			Univariate	Multivariate		
		P	HR	95% CI	P	P	HR	95% CI	P
Gender									
Female	51								
Male	95	0.654				0.432			
Age (y)									
<65	86					0.583			
>=65	60	0.138							
Distance from anal verge (cm)									
<=5 (low)	58								
<=10 (middle)	88	0.280				0.197			
Neoadjuvant therapy									
No	88								
Yes	58	0.692				0.637			
CEA									
<5	92								
>=5	54	0.972				0.357			
CA19–9									
<37	131								
>=37	15	0.076				0.583			
MR-DME (mm)									
<=4	47		1				1		
>4	99	**0.031**	2.090	(0.566, 7.713)	0.268	0.333			
MRF									
Negative	117								
Positive	29	0.172				0.938			
MR-LN									
Negative	86		1				1		
Positive	60	**0.009**	1.443	(0.578, 3.602)	0.432	0.067			
MR-EMVI score									
0–2 (negative)	109		1				1		
3–4 (positive)	37	**0.001**	**3.236**	**(1.328, 7.885)**	**0.010**	0.263			

DFS: Disease-free survival OS: Overall survival HR: hazard ratio.

CI: confidence interval MRF: mesorectum fascia.

**Table 3:** The survival analysis according to MRI-detected factors.

Characteristics	No. of Patients	3-year DFS(%)	χ^2^	HR	95% CI	P	3-year OS(%)	χ^2^	HR	95% CI	P
All of patients	146	84.9	—	—	—	—	95.6	—	—	—	—
MR-EMVI Positive **VS** Negative	37 VS 109	60.3 VS 86.2	12.11	3.557	2.028–13.32	<0.01	87.8 VS 96.3	1.25	2.292	0.479–14.90	0.26
MR-DME > 4 mm **VS** <= 4 mm	99 VS 47	75.7 VS 89.8	5.37	3.744	1.165–5.992	0.02	92.7 VS 100.0	2.71	—	—	0.10
MR-LN Positive **VS** Negative	60 VS 86	69.9 VS 87.2	7.62	2.946	1.386–6.699	<0.01	89.1 VS 98.8	4.65	7.432	1.180–30.87	0.03
MR-EMVI Positive and MR-DME > 4 mm **VS** MR-EMVI Negative and MR-DME <= 4 mm	32 VS 42	60.7 VS 93.3	14.88	10.04	3.110–32.41	<0.01					
MR-EMVI Positive and MR-LN Positive **VS** MR-EMVI Negative and MR-LN Negative	24 VS 74	57.2 VS 90.8	16.54	14.88	1.050–54.68	<0.01

## Discussion

To the best of our knowledge, this is the first study that combining MR-EMVI, MR-DME, and MR-LN to predict the prognosis of patients with clinical T3 mid-low rectal cancer. These three important characteristics play a key role in predicting the DFS. Furthermore, MR-EMVI was the only independent prognostic factor.

Venous invasion detected in the histopathological examination was associated with a higher incidence of local or distant recurrence^2–7^ and poorer overall survival rate^28^. It is accepted that venous invasion allows tumor cells to embolize by means of the hematogenous spread. As a result, distant metastasis in rectal cancer, via portal circulation, is formed^29^.

The incidence of MRI-detected EMVI-positive rectal cancer in the present study was 25.3% (37/146), which is similar to previous study (15.3–65.5%)^17,21,30–32^. Several studies had found the relevance between MR-EMVI and prognosis^21,30–33^. These results revealed that MR-EMVI significantly correlated with DFS, OS, and risks of synchronous^5^ and or metachronous^30^ metastasis. Our study also showed that there was significant difference in the incidence of metastasis among rectal cancer patients with or without positive MR-EMVI. Consequently, patients with MR-EMVI score ≥ 3 had a significantly worse DFS (p < 0.01).

On the other hand, many authors had reported a prognostic influence of the mesorectal extension^8–16^. As the DME becomes deeper, it is considered that more undetectable lymphovascular invasions existed in the mesorectal adipose tissue^13^. With regard to the cut-off value of the MR-DME, different studies adopted different cut-off value to subdivide the MR-DME and these studies also showed different prognosis^8,10–16,34,35^. Shirouzu *et al*.^13^ and Akagi *et al*.^36^ recommended that a value of 4 mm as the optimal cut-off value to predict oncologic outcomes. Thus, we adopted 4 mm as the cut-off value for the MR-DME classification in our study. Moreover, our results showed that this classification was associated with the 3-year DFS.

Furthermore, when combining MR-EMVI with MR-DME or MR-LN, the prognostic significance was more remarkable. Patients with MR-EMVI score ≥ 3 and MR-DME > 4 mm had a significantly worse 3-year DFS than those with MR-EMVI score ≤ 2 and MR-DME < 4 mm (p < 0.01) (Fig. 9A). The oncological outcomes were similar for patients with MR-EMVI score ≥ 3 and positive MR-LN (p < 0.01) (Fig. 9B). According to the multivariate analysis, the MR-EMVI score had a significant impact on the 3-year DFS (p = 0.01). Therefore, we believed that MR-EMVI could be considered as an important prognostic factor and combining MR-EMVI with MR-DME or MR-LN could improve the accuracy of predicting the prognosis of patients with clinical T3 mid-low rectal cancer before surgery.

However, the positive or negative MRI-EMVI and the MR-DME, as well as MR-LN status does not directly influence preoperative treatment regimens currently. Given that the poor prognosis associated with these prognostic factors, it is necessary to assess MR-EMVI, MR-DME and MR-LN status preoperatively to predict high-risk patients of recurrence and prognosis. For these patients, neoadjuvant treatment was needed to eradicate tumor cells in the circulation and lymphovascular invasion concealed in the mesorectal to prevent postoperative recurrence and improve survival.

This retrospective study had some limitations. First, the inclusion criteria may cause selection bias because patients who did not have an MRI before operation were excluded. Second, although the radiologists were blinded to the clinicopathological featuresand survival outcomes, it was impossible to blind them to other imaging characteristics of rectal cancer, which might have an effect on reported findings. Third, the accuracy of MRI-related data depended on the radiologists’ experience. Furthermore, this study was carried out in one single center and the number of patients was too small to draw a definitive conclusion.

## Conclusions

The MR-EMVI, MR-DME, and MR-LN were important preoperative prognostic factors for patients with clinical T3 mid-low rectal cancer, and the MR-EMVI is an independent prognostic risk factor. Preoperative MR-EMVI combing with MR-DME or MR-LN status can improve the accuracy of predicting prognosis of clinical T3 mid-low rectal cancer. These preoperative features could be used to guide treatment pathways to improve prognosis. In future, more prospective multi-center large sample studies were desired to confirm these findings.
